# Multiomics Approaches for Bacteriophage‐Based Biocontrol Applications: A Review of Metabolomics, Transcriptomics, and Proteomics

**DOI:** 10.1155/bmri/8304153

**Published:** 2026-05-06

**Authors:** Daniel Jesuwenu Ajose, Collins Njie Ateba

**Affiliations:** ^1^ School of Biology and Environmental Sciences, Faculty of Agriculture and Natural Sciences, University of Mpumalanga, Mbombela, South Africa, ump.ac.za; ^2^ Food Security and Safety Focus Area, Faculty of Natural and Agricultural Sciences, North-West University, Mmabatho, South Africa, nwu.ac.za

**Keywords:** antibiotics, bacteria–phage interaction, drug resistance, multiomics, next-generation sequencing, phage therapy

## Abstract

The emergence of multidrug‐resistant (MDR) in various microorganisms due to prolonged antibiotic treatment poses a growing worldwide health issue. It is essential to identify alternative, effective methods to address MDR bacterial diseases, as treating infections caused by these pathogens can be challenging. Bacteriophages, also known as phages, are viruses that target and destroy bacteria. They are being investigated and utilized as alternatives to existing therapies because of their effectiveness and specificity. Phages are often considered safe substitutes for antibiotics because they naturally occur in the environment. Phage replacement therapy involves complex biological processes driven by interactions between phages and bacteria. An integrative approach is essential for a comprehensive analysis of these processes and for understanding the relationships between different biomolecules and their functions. This includes combining data from various omics, such as transcriptomics, proteomics, and metabolomics. High‐throughput technology has revolutionised research, enabling the development of numerous tools and methods for integrating and interpreting multiomics data from diverse samples. This paper presents an overview of omics technologies and highlights strategies for integrating them across layers. By comparing data from multiomics and single‐omics studies, a deeper understanding of the information flow underlying phage therapy will be gained.

## 1. Introduction

The discovery of antibiotics stands as one of the most pivotal medical advancements of the 20th century, serving as an essential instrument in the treatment and prevention of diseases affecting both humans and animals [1, 2]. Nevertheless, the careless application of antibiotics across various domains of One‐Health, coupled with concerns about antibiotic resistance in bacterial pathogens, has sparked heightened interest in imposing global restrictions on antibiotic use. Given the shortcomings of traditional antimicrobial agents, it is noteworthy that only 15 novel antibiotics have been introduced clinically since 2000. Furthermore, there have been accounts of resistance to certain newly developed antibiotics. This has prompted an exploration of alternative strategies, including phage therapy, to address the challenge posed by antimicrobial‐resistant pathogens [1, 3].

Phage therapy is the use of bacteriophages (phages), viruses that selectively infect and eliminate bacteria, as a therapeutic approach for bacterial illnesses. This strategy holds significant importance in combating antibiotic‐resistant microorganisms [4, 5]. The isolation of phages from natural habitats or their engineering to selectively target bacterial strains enables a more individualized approach to therapy. Phages exhibit a high degree of specificity towards their bacterial targets by attaching to bacterial cells, introducing their genetic material, and interacting with the bacteria’s machinery to undergo replication. This process ultimately results in the annihilation of the bacterial cell [6]. The high specificity of phages enables them to selectively target pathogenic bacteria while preserving the integrity of beneficial bacteria, providing a notable advantage over broad‐spectrum antibiotics. Many bacteria have developed resistance mechanisms against traditional antibiotics, rendering these therapies obsolete. With the ongoing increase in antibiotic resistance, phage therapy offers a highly promising option [7].

Although phage therapy has a lengthy history tracing back to the early 20th century, it has recently regained prominence due to the escalating issue of antibiotic resistance [1, 8]. Contemporary progress in omics technologies and biotechnology has enhanced our capacity to create and implement phage therapeutics, despite the concept not being novel [9, 10]. Ongoing clinical trials and research are being conducted to advance our understanding of the efficacy and safety of phage therapy in treating various infections [11–13]. An essential aspect of optimizing and refining this therapeutic strategy is understanding the fundamental biological processes at multiple levels.

The multiomics approach is a general methodology in biomedical and life sciences research that combines data from multiple “omics” disciplines to obtain a comprehensive and integrated understanding of biological systems [14, 15]. “Omics” refers to the field of large‐scale investigations of biological molecules. The multiomics approach integrates data from several omics layers, including genomes, transcriptomics, proteomics, and metabolomics, to offer a comprehensive understanding of the functioning and environmental responses of biological systems [16]. Genomics involves the quantitative analysis of an organism’s entire DNA, encompassing both its sequence and structure [17]. Transcriptomics, on the other hand, focuses on the comprehensive collection of RNA transcripts generated by the genome, providing valuable insights into gene expression and control in the context of phage therapy. Analysis of RNA expression, known as transcriptomics, can reveal alterations in gene expression and shed light on phage effects on bacterial regulatory networks, aiding the development of more sophisticated phage formulations [18]. In proteomics, researchers undertake the comprehensive analysis of proteins produced by an organism, exploring their functions, interactions, and alterations [19]. Proteomics enables a comprehensive examination of all proteins in an organism, providing valuable insights into protein expression and alterations. Understanding these protein dynamics can help advance phage therapeutics with improved selectivity and effectiveness. Molecular metabolomics involves examining metabolic profiles to understand the biochemical alterations and metabolic conditions in bacteria when exposed to phage therapy [20].

Through the integration of diverse omics data, comprehensive knowledge on the strategies through which genetic molecules manifest their functional outcomes at the molecular, cellular, and systemic scales can be derived [21, 22]. This holistic perspective clarifies intricate biological processes, disease mechanisms, and individual genetic variations in relation to food, health, and disease. An investigation into phage resistance in the food industry using multiomics analysis revealed that the development of phage resistance has a pleiotropic effect on several cellular processes and metabolism [23]. This analysis offers a more comprehensive evaluation and understanding of the impact of phage candidate development on resistance [23]. Phages have been used in clinical settings, especially in Eastern Europe, for a considerable period [24]. In its early years, phage therapy was hindered by a lack of understanding of phage biology. Additionally, current phage therapy faces challenges, including safety and regulatory issues, across various regions, including the United States and Western Europe [25]. This review highlights the innovative applications of metabolomics, transcriptomics, and proteomics in enhancing the effectiveness of phage therapy. By integrating data from metabolomics, transcriptomics, and proteomics, this review is aimed at providing a comprehensive understanding of how various omics methodologies can enhance the effectiveness of phage therapy. Utilizing this holistic approach can lead to more effective phage designs, better predictions of treatment effectiveness, and ultimately, more successful applications of phage therapy in combating bacterial diseases.

### 1.1. Method for Selection of Literature

Data was retrieved from scientific databases such as Google Scholar, PubMed, Scopus, ScienceDirect, and Web of Science. Keywords or search strings used in the search include phage therapy, multi‐omics, transcriptomics, metabolomics, proteomics, genomics, antibiotics, bacteria–phage interactions, omics, and phage. These keywords were used alone and in combination to find relevant material from electronic databases. To be considered for inclusion in the review, peer‐reviewed studies, official reports, and policy documents relevant to infection prevention and AMR included and identified information about the application of phage therapy and omics bioinformatics tools. To ensure currency and relevance, results were filtered by publication date (2015–2025), language (English only), and access type (open‐access articles). Articles that did not meet these criteria were excluded.

## 2. Phage Therapy: An Overview

Phage therapy, the use of bacteriophages for treating bacterial infections, has gained renewed attention recently as a potential solution to the growing problem of antibiotic resistance [26, 27]. Phage therapy, a notion initially developed in the early 20th century, lost popularity with the introduction of antibiotics [28]. Bacteriophages provide a natural alternative to current antibiotics [29]. The prevalence of multidrug‐resistant (MDR) bacteria has attained an incongruous level worldwide and threatens global public health as a silent pandemic, thus leading to a revival in phage research and its therapeutic applications [3].

Phages function by binding to unique receptors on the surface of bacterial cells, introducing their genetic material into the host, and commandeering the bacterial machinery to generate new virions [30]. This process ultimately leads to the lysis or destruction of the bacterial host cell, thereby liberating new phages that can also infect additional bacterial cells. Phage therapy is no longer a futuristic medical concept but rather a real, life‐saving intervention for patients suffering from severe MDR bacterial infections [27, 31, 32]. The specificity of phages to bacterial hosts is both a benefit and a constraint to phage therapy [33]. Although it enables precise treatment with little interference with the body’s indigenous microbiota, it limits the potential to infect multiple strains that may be present in each disease situation [34]. Moreover, the need to better understand phage–bacteria interactions is also critical to ensure the success of phage therapy applications [5, 35]. Table 1 provides an overview of some documented phage applications.

**Table 1:** Recent case studies of the application of phages.

**Clinical application**				
**Causal agent**	**Infection caused**	**Route of administration/methodology**	**Outcome/result**	**Reference**

XDR *Acinetobacter baumannii* and multidrug‐resistant (MDR) *Klebsiella pneumoniae*	Bone infection caused by trauma	Intravenous	Quick tissue repair and elimination of pathogens after 35 weeks	[36]
MDR *Pseudomonas aeruginosa*	Cystic fibrosis (CF)	Intravenous	No more episodes of CF flare‐ups after about 14 weeks	[37]
Penicillin‐resistant *Staphylococcus aureus*	Osteomyelitis in diabetic foot ulcers	Soft tissue	Full recovery following a 3‐year period	[38]
MDR *Pseudomonas aeruginosa*	Septicaemia	Intravenous	Although the patient’s blood was disinfected by phage therapy, the administration of the medication had to be stopped due to escalating heart failure and allergy risk	[39]
MDR *Pseudomonas aeruginosa* and *Staphylococcus aureus*	Hip infection	Soft tissue	No clinical indications of illness after one and a half years	[40]
MDR *Acinetobacter baumannii*	Necrotizing pancreatitis	Intravenous and soft tissue	Recovery from infection and restoration of health	[41]
*Staphylococcus aureus*	Long‐term skin illness	Topical application of phage preparation/oral	Decrease in the number of bacteria and improved clinical state	[42]
Colistin‐only‐sensitive *Pseudomonas aeruginosa*	Bacterial blood infection and knee infection	Intravenous and soft tissue	Septicaemia cleared; however, the pathogen was still present in the wound	[43]
MDR *Escherichia coli*, *Staphylococcus aureus*, *Klebsiella pneumoniae*, *Enterobacter cloacae*, *Klebsiella aerogenes*, *Pseudomonas aeruginosa*, and *Enterococcus faecium*	Various infections, including bacteremia, osteomyelitis, joint, urinary tract, and respiratory infections	Several routes, including intravenous, topical, intra‐articular, intraoperative	66% of patients showed favorable responses; 42% achieved bacterial eradication; and no major adverse reactions were reported	[44]
*Staphylococcus aureus*	Diabetic foot infection	Topical	Anti‐*S*. *aureus* phage therapy was administered to 10 high‐risk amputation patients. The treatment was effective in 9 patients; 1 patient did not respond	[45]
*Klebsiella pneumoniae*	Sternal wound infection (mediastinitis) following aortic arch surgery	Intravenous	The patient remains infection‐free after 2 years of follow‐up intravenous and local administration	[46]
*Staphylococcus epidermidis*	Chronic orthopedic infection	Intraoperative	Phage therapy administered with a successful outcome. The patient remained infection‐free at the 6‐month follow‐up	[47]

**Application in foods**				
**Phage**	**Product tested**	**Methodology**	**Outcome**	**Reference**

phiEco1, phiEco2, phiEco3, phiEco5, phiEco6, and phiS1	Oyster	A 24‐h‐old bacterial culture was added to the oysters and incubated for 1 h at 37°C to allow attachment. The oyster meat was then incubated appropriately after the addition of the phage suspension	Phages were still able to lower the *E*. *coli* concentration on the oyster meat	[48]
Cocktail composed of phages DT1–DT6	Milk and meat	A phage cocktail was applied to samples under various circumstances	At both 24°C and 37°C, the phage cocktail effectively reduced the *E*. *coli* burden in the food products tested	[49]
*E*. *coli*‐specific phage	Lettuce, cucumber, and carrot	The various veggies were submerged in *E*. *coli* suspensions for 30 min, and then, they were kept at 37°C for 24 h. After that, the veggies received successive treatments from phages and cold nitrogen plasma	After 9 days of the sequential treatment, no viable bacteria were found. On the 3rd day, cell viability had decreased to 1.21 log10 CFU/g. The temperature difference had no bearing on the outcomes	[50]
Phage OSY‐SP	Green pepper and spinach	*E*. *coli* was spot‐injected into both matrices (pepper and spinach). To optimize the phage application process, the products were rinsed with phage lysate and phages suspended in PBS prior to storage	Cell loss was noted under chilled storage circumstances. In both fresh produce tests, the phage rinse treatment was effective	[51]
FAHEc1	UHT milk; ready‐to‐eat meat; raw beef	Phages and *E*. coli O157:H7 were added to UHT milk for inoculation. To mimic the phage application immediately prior to slathering in carcasses, raw beef was inoculated at 37°C	UV‐treated phages (UVPs) reduced the number of colony‐forming units (CFUs) by 2–2.5 log10 in milk and 1.75–2.5 log10 in raw beef	[52]

Abbreviations: MDR, multidrug‐resistant; XDR, extensively drug‐resistant.

## 3. Multiomics in Phage Therapy

To gain a comprehensive understanding of biological systems, the multiomics approach combines data from various “omics” disciplines, such as genomics, transcriptomics, proteomics, and metabolomics [53]. In the field of phage therapy, omics techniques are particularly beneficial as they allow researchers to explore the complex interactions between bacteriophages and their bacterial hosts at multiple molecular levels [23, 54]. Examining these interactions from different omics perspectives offers opportunities to gain valuable insights into the effects of phages on bacterial gene expression, protein synthesis, and metabolic pathways [55]. This knowledge is crucial for optimizing phage therapy, as it helps in identifying key weaknesses in bacteria that phages can exploit. Moreover, the utilization of a multiomics methodology could enhance the development of individualized phage therapeutics tailored to the specific characteristics of a disease, potentially improving therapeutic outcomes [56–58]. Therefore, the integration of multiomics data holds great promise in enhancing the efficacy and precision of phage therapy.

### 3.1. The Value of Multiomics in Phage Therapy

The dynamic interactions between phages and their bacterial hosts make phage therapy a complex process, despite its potential [5, 26]. Conventional approaches, which focus on discrete elements like gene expression or protein synthesis, may provide only a partial understanding of these interactions. In contrast, bacteria respond to phage infection simultaneously on multiple levels, including genetic, transcriptomic, proteomic, and metabolic responses. The multiomics approach, which integrates these different layers, enables a more comprehensive understanding of how phages operate within bacterial cells and how bacteria can develop resistance [35, 59]. For instance, the translation of stress reactions by bacteria into functional proteins can be uncovered by combining transcriptomic data, which tracks changes in gene expression, with proteomic data, which shows alterations in protein quantity and modification [60]. On the other hand, metabolomic data can illustrate how these proteins impact metabolic pathways, aiding the bacterium in survival, or how the phage disrupts these pathways, leading to bacterial lysis [61]. Omics technologies have therefore been successfully employed to accurately characterize bacteriophages and their genes and proteins important for interaction with bacterial hosts [62].

### 3.2. Benefits of Integrating Omics Data

To understand the molecular mechanisms by which phages hijack their bacterial hosts, omics technologies have the potential to provide novel insights into the organization of transcriptional and translational events that occur during the infection process [63]. Integrating multiomics datasets enables the identification of key points in bacterial metabolism or regulatory networks that phages exploit [22]. This data is essential for developing more efficient phage‐based treatment agents that can simultaneously target several pathways with a significant decrease in bacterial resistance [23]. More so, multiomics methodologies allow for the development of highly sensitive pathogen‐targeted phage‐based treatments, resulting in more efficient biocontrol or therapeutic procedures [64, 65]. The potential of multiomics approaches to provide information on gene expression, uncover the appropriate quantities and conformations of proteins, and elucidate the impact of these proteins on the metabolic functions within the cell makes multiomics protocols a unique strategy in obtaining insights into the mechanisms underlying phage–bacteria interactions [22, 66].

### 3.3. Challenges of Multiomics Approaches and Future Perspectives

Notwithstanding its potential, the multiomics method presents some difficulties as the integration of extensive and intricate datasets from many omics disciplines requires sophisticated computational tools and specialized knowledge in bioinformatics [67, 68]. Furthermore, the analysis of multiomics data is complex, since alterations documented at one molecular level may not exhibit a direct correlation with those at another level. As such, advancements in computational approaches and machine learning algorithms have the potential to enhance the capacity to combine and interpret multiomics data, hence becoming a more reliable tool in phage therapy research [69–71]. Additionally, the multiomics approach provides a comprehensive framework for understanding and enhancing phage therapy. The integration of data from metabolomics, transcriptomics, proteomics, and other omics disciplines enables researchers to create more efficient, focused, and individualized phage therapies, potentially transforming the battle against antibiotic‐resistant bacteria [65].

## 4. Metabolomics in Phage Therapy

Understanding the biochemical alterations brought about by phage infection can be achieved with metabolomics, the study of the complete set of tiny molecules or metabolites within a biological system [72, 73]. The concentrations of metabolites, as byproducts of cellular activities, provide a real‐time representation of a cell’s physiological state. The application of metabolomics in phage therapy significantly enhances our understanding of the interference of phages with bacterial metabolism, as well as the mechanisms by which bacteria develop resistance to these biocontrol agents [74].

### 4.1. Analytical Approaches in Metabolomics

Metabolomic analysis commonly employs mass spectrometry (MS) and nuclear magnetic resonance (NMR) spectroscopy methods to accurately measure and categorize metabolites in a sample [75, 76]. These methodologies can be used to examine the metabolite profiles of bacteria before and during phage infection, thereby uncovering alterations in metabolic pathways that may be crucial for the survival or death of the bacterial cell [77, 78].

Both targeted and untargeted metabolomics offer unique benefits in phage research. Targeted metabolomics emphasizes specific metabolites, ensuring elevated sensitivity, precision, and dependable quantitative results [79]. This is particularly advantageous for examining specific hypotheses about host–phage interactions, such as nucleotide pools during phage replication [80, 81]. Nevertheless, its scope is constrained, and it may overlook unforeseen or innovative metabolic alterations triggered by infection.

In contrast, untargeted metabolomics encompasses a wide array of metabolites, facilitating the identification of previously unrecognized pathways that are modified during phage infection [81]. This holds significant importance considering the intricate nature and swift metabolic reorganization induced by phages. The compromise involves reduced quantitative accuracy, increased complexity in data analysis, and possible difficulties in metabolite identification [79, 82].

Phage infection frequently triggers rapid, transient alterations in metabolite flux, making quenching essential to maintain an accurate representation of intracellular conditions [83]. Efficient quenching techniques, such as swift cooling with cold solvents or filtration followed by freezing, promptly halt enzymatic activity within moments, thereby averting metabolic alterations postsampling [84]. In the absence of adequate quenching, quantified metabolite concentrations may fail to accurately reflect in vivo conditions, thereby compromising the integrity of both targeted and untargeted analyses.

### 4.2. Role and Impact of Metabolomics in Phage–Bacteria Interactions

Phage infection induces substantial metabolic changes in the bacterial cell, resulting in the production of additional virions. These changes can encompass modifications in energy generation, nucleotide synthesis, and membrane lipid metabolism, all of which are essential for the propagation of DNA viruses [85]. A few studies have demonstrated that phage infection results in an augmentation in the synthesis of specific nucleotides and amino acids that are crucial for the formation of phage DNA and proteins [83, 86]. By identifying these important metabolic alterations, metabolomics can provide prospective targets for improving phage therapy [87]. Phages that exploit specific metabolic pathways during infection of the host cell and result in bacterial lysis may be very useful in devising treatments that further diminish these pathways, hence increasing the vulnerability of the bacterial cell to the phage. In general, metabolomics plays a crucial role in phage therapy by providing in‐depth knowledge that enables the development of methods to enhance the specific selection of phages, techniques to increase the susceptibility of bacteria to phages, and a potential decrease in bacterial resistance to phages [62]. As the field of metabolomics advances, its integration with other omics methodologies is expected to yield more advanced and efficient phage‐based therapies.

### 4.3. Case Studies in Metabolomics and Phage Therapy

The propagation of phages depends on bacterial metabolism; thus, several studies have reported the significance of metabolomics in enhancing phage therapy [88, 89]. A pertinent example is the examination of *Pseudomonas aeruginosa*, a prevalent microorganism associated with diseases acquired in healthcare facilities [90]. Analysis of the metabolome of *P. aeruginosa* after phage infection showed notable alterations in its core carbon metabolism, particularly in the tricarboxylic acid (TCA) cycle and glycolytic pathways [90]. Through the analysis of these metabolic changes, certain metabolic obstacles that may be utilized to improve the effectiveness of phages were successfully detected. A further illustration is the utilization of metabolomics to comprehend the processes of phage resistance in bacteria. The identification of metabolic alterations that provide resistance to phages can offer valuable insights into strategies to counteract or prevent the development of such resistance [91–93].

## 5. Transcriptomics in Phage Therapy

The examination of RNA transcripts produced by the genome, referred to as transcriptomics, is crucial for understanding the complex changes in gene expression that occur during phage infection of bacterial cells [85]. Through the examination of the transcriptome, it is possible to identify the specific genes that are activated or deactivated in response to phage attack [94]. This analysis provides a valuable understanding of bacterial defense mechanisms and the strategies employed by phages to hijack the cellular machinery of their host.

### 5.1. Techniques and Methodologies in Transcriptomics

For transcriptomic investigations, high‐throughput sequencing methods such as RNA‐Seq are used to comprehensively profile all RNA molecules in a cell, including mRNA, rRNA, tRNA, and noncoding RNAs [95, 96]. The aforementioned methodology facilitates the identification of alterations in gene expression during several phases of phage infection, encompassing the first adherence of the phage to the bacterial cell and subsequent cell lysis [62]. RNA‐Seq provides a detailed assessment of the transcriptome, including precise measurements of gene expression levels and uncovering alternative splicing processes, posttranscriptional changes, and the presence of hitherto unidentified genes [97, 98]. The provided insights are of great value for analyzing the intricate regulatory networks that control the interactions between phages and bacteria.

### 5.2. Role and Significance of Transcriptomics in Understanding Phage–Bacteria Interactions

Phage infection induces significant alterations in the transcriptome composition of the bacterial host. The field of transcriptomics offers a thorough perspective on the alterations in gene expression that take place during phage infection, therefore providing insight into the molecular mechanisms utilized by both phages and their bacterial hosts [99]. During the initial stages of infection, phages frequently stimulate the production of their own genes while concurrently deactivating the host’s transcriptional control system. An example is the transcriptomic study of *Escherichia coli* infected by T4 phage, which documented a swift decrease in host genes related to DNA replication and repair, as well as an increase in phage genes essential for DNA packaging and phage assembly [63]. This subversion enables phages to give priority to the synthesis of viral components required for the formation of new virions [18] and to enhance phage therapy methods, such as modifying phages to bypass bacterial defense mechanisms or combining phages with adjuvants that selectively block bacterial pathways.

Transcriptomics can reveal the bacterial stress responses elicited by phage infection, including the activation of SOS responses, a regular regulatory network employed by bacteria to repair DNA damage [100, 101]. A proper understanding of these reactions is crucial for developing effective strategies to combat bacterial resistance to phages. Integrating transcriptome data with other omics datasets, such as proteomics and metabolomics, allows for a comprehensive understanding of the phage–host interaction, thereby facilitating the development of more efficient and tailored phage‐based treatments [54].

### 5.3. Case Studies in Transcriptomics and Phage Therapy

Several studies have applied transcriptomics to phage therapy, and a classic example is the investigation of *Vibrio cholerae*, the causative agent of cholera, during infection with a lytic phage, denoted International Centre for Diarrheal Disease Research, Bangladesh cholera phage 1 (ICP1 phage) [102–104]. Transcriptomic studies have demonstrated that this phage commandeers the host’s RNA polymerase, diverting its activity to transcribe genes specific to the phage while suppressing the expression of host‐relevant genes [102]. This provided a detailed understanding of how the ICP1 phage effectively regulates the bacterial transcriptional apparatus to guarantee its successful spread within the host cell, as well as its contribution to the evolution of epidemic *V*. *cholerae*.

Additionally, transcriptomic profiling of *P. aeruginosa* during phage infection revealed the activation of genes associated with oxidative stress and DNA repair, indicating the host cell’s efforts to mitigate damage caused by phages [105, 106]. These, therefore, indicate that phage therapy can be optimized to increase bacterial sensitivity to phage attack by carefully targeting these stress response pathways [35].

## 6. Proteomics in Phage Therapy

A crucial tool in the field of protein‐level interactions between bacteriophages and their bacterial hosts is proteomics, which is the comprehensive study of proteins within a biological system [107]. Analyzing changes in the proteome during phage infection provides a direct understanding of the mechanisms through which phages affect bacterial cells and how bacterial hosts react to these attacks, since proteins are the primary functional molecules carrying out most cellular processes [63, 108].

### 6.1. Techniques and Methodologies in Proteomics

Proteomic analysis commonly employs methods such as MS to detect and quantify proteins in complex mixtures and two‐dimensional gel electrophoresis (2D‐GE) to separate proteins based on their respective isoelectric points and molecular weights [109]. Progress in MS, including tandem MS/MS and label‐free quantification techniques, has greatly enhanced the sensitivity and precision of protein identification [110]. This has enabled scientists to analyze hundreds of proteins in a single operation. These methods enable the detection of phage‐encoded proteins produced during infection, as well as bacterial proteins whose expression may be altered during phage invasion [111]. Careful analysis of the proteome profiles of bacterial cells infected with phages and those that are not infected enables the mapping of protein‐level alterations that occur during the various phases of phage infection.

### 6.2. Role of Proteomics in Understanding Phage–Bacteria Interactions

The field of proteomics offers comprehensive data on the posttranslational modifications (PTMs) of proteins, including phosphorylation, acetylation, and ubiquitination, which have the potential to greatly impact protein function and stability [112]. PTMs are frequently very crucial in controlling the function of both bacterial and phage proteins during phage infection [113]. For instance, certain phages encode proteins that selectively alter bacterial proteins to interfere with regular cellular functions or to avoid bacterial defenses [114].

Evidence from proteomic investigations has shown that phage infection can cause the targeted breakdown of bacterial proteins, inhibition of bacterial defense mechanisms, and alteration of host cellular pathways to promote viral reproduction [86, 74]. An investigation of *Staphylococcus aureus* infected by the phage P68, using proteome analysis, revealed the breakdown of host proteins responsible for DNA repair and metabolic pathways [115]. The degradation significantly reduced the bacterial host cell’s ability to recover from the damage caused by the phage [115].

### 6.3. Case Studies in Proteomics and Phage Therapy

Several studies have assessed the impact of proteomic investigations on phage–host interaction that may be useful in the development of more successful therapeutic applications for phages [63, 116, 117]. An exemplary demonstration of proteomics in phage therapy is the study of *Listeria monocytogenes* infected by the A511 phage. The study accurately detected the expression of phage proteins that inhibit bacterial cell wall and protein synthesis and efficiently trigger bacterial lysis. These findings suggest potential focal points for either developing phages with enhanced lytic capabilities or improving their bactericidal action [116]. In a separate investigation, proteomics was used to detect bacterial proteins that are either reduced in expression due to phage hijacking or increased in expression to promote defense by *E*. *coli* infected with T4 phage. The investigation identified crucial proteins involved in maintaining the cell membrane’s integrity and responding to stress, thereby offering insights into the potential exploitation of phage therapy [63].

### 6.4. Impact of Proteomic Data on Phage Therapy

In addition to identifying potential indicators of bacterial sensitivity to phages, proteomics provides a more comprehensive understanding of how phages regulate bacterial cells. By quantifying the proteome of bacteria during phage infection, it is possible to pinpoint crucial proteins or pathways that, when interfered with by phages, lead to bacterial death [118]. This knowledge can be applied to develop more efficient phage therapies, such as combining phages with drugs that target bacterial proteins or modifying phages to directly transport harmful proteins to bacterial cells [119].

Furthermore, combining proteomics with other omics techniques can provide a better understanding of how phages and hosts interact, thus leading to the discovery of the complex networks that control bacterial survival and phage effectiveness [70]. Future advancement of personalized phage therapeutics, tailored to the distinct proteomic and metabolic characteristics of bacterial pathogens in individual patients, is anticipated to be greatly impacted by this multiomics methodology [66, 67].

## 7. Integration of Multiomics Data in Phage Therapy

Multiomics‐based data on bacteria during phage infection provide a system‐level understanding of how phages subvert the cellular machinery of their target hosts [120]. The integration of knowledge from transcriptomics, proteomics, metabolomics, and other omics fields offers a state‐of‐the‐art approach to phage therapy and a comprehensive understanding of phage–host interactions [121]. This approach also affords the opportunity to overcome the limitations of single‐omics studies, which can only provide a limited understanding of the numerous biological pathways involved in phage infection.

### 7.1. Importance of Multiomics Integration

**Figure 1:**
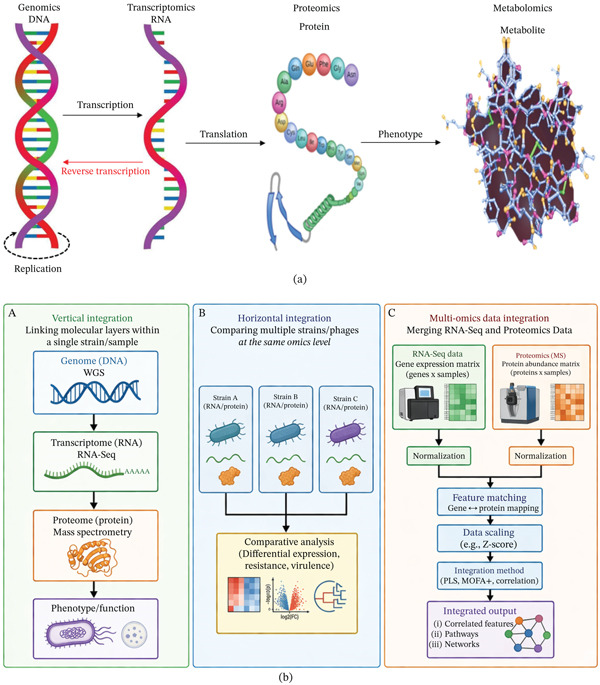
(a) Overview of multiomics approaches. (b) Diagrammatic illustration of strategies for the integration of multiomics data. Vertical integration connects the genomic, transcriptomic, and proteomic layers within a unified framework. (B) Horizontal integration involves comparing omics data across strains or phages. (C) The synthesis of RNA‐Seq and proteomics data demonstrates the processes of normalization, feature matching, scaling, and multivariate analysis to produce outputs that hold biological significance.

The molecular alterations that occur within bacterial cells during phage infection are analyzed from a different angle by each field of omics. Proteomics reveals changes in protein levels and modifications, metabolomics highlights changes in metabolic pathways, and transcriptomics explains variations in gene expression. These facts are related, though, since the genes are translated into RNA, RNA is translated into proteins, and proteins accelerate metabolic processes [122], which is the fundamental view [123] (Figure 1a). Researchers may create intricate models of how phages affect bacterial cells and how bacteria respond by combining data from several sources across multiple levels (Figure 1b).

For example, correlations between transcriptome alterations and related proteomic shifts, such as the upregulation of RNA transcripts resulting in higher protein production, can be identified using a multiomics method [124]. In a similar vein, combining metabolomic data can shed light on how these modifications to the protein impact bacterial metabolism, offering a more comprehensive understanding of the physiological effects of phage infection [125].

### 7.2. The Role of Multiomics Integration in Precision Phage Therapy Development

The integration of multiomics data has significantly advanced the development of phage therapy [23, 54]. This technology has enabled the identification of essential bacterial processes that phages target, thereby improving the development of phages that target multiple pathways simultaneously, thus reducing the likelihood of bacterial resistance development. Furthermore, the use of multiomics approaches has facilitated the development of more tailored phage therapies, in which phages are selected or altered based on the distinct molecular features of the pathogen in a particular patient [126].

Moreover, the identification of biomarkers that predict the efficacy of phage therapy relies on the integration of several omics techniques [126]. Researchers can develop diagnostic techniques to choose the most effective phages for treating infections by identifying well‐defined metabolic or proteomic pathways associated with successful phage infection [77, 127].

### 7.3. Applications of Multiomics in Phage Therapy

Literature has a few omics‐acclaimed approaches in phage research. Table 2 provides an overview of these approaches, which include their potential as biocontrol agents, protein profiling, and changes in phage–host interactions, such as transcriptional responses and active gene profiling.

**Table 2:** Overview of multiomics approach in phage research.

Phage	Host	Omics technique	Application	Major finding	Reference
Phage cocktail	*P*. *aeruginosa*	Metabolomics	Modifications in the metabolic functioning of the host during infection	Rapid, dynamic metabolic shift in the host	[90]
Dp‐1 and Cp‐1	*S*. *pneumoniae*	Proteomics	Phage–host interaction	System‐level (highly specific, nonrandom protein–protein interactions) view of phage–host	[128]
PaP3	*P. aeruginosa*	Transcriptomics	Phage–host interaction	Dynamic, bidirectional interaction between the phage and the host	[129]
vB_BpuM_BpSp	*Bacillus*	Proteomics	Specific protein analysis	Identified the actual structural protein composition of a jumbo phage, including novel functional proteins	[130]
MC1061 (*ϕ*24_B_)	*E*. *coli*	Transcriptomics	Modified host function upon phage infection	Revealed functional consequences of lysogeny at the regulatory level	[131]
*Φ*MSP	*S*. *aureus*	Proteomics	Protein analysis	Identified the structural protein composition of lytic *Staphylococcus aureus* phages	[132]
T5‐like phage	*E*. *coli* O157:H7	Genomics, proteomics	Biocontrol	Validated functional expression	[133]
*ϕ*TMA	*Thermus thermophilus*	Genomics, proteomics	Genome and protein profiling	Validated genome annotations and assigned functional relevance	[134]
*Φ*CP39O and *Φ*CP26F	*Clostridium perfringes*	Genomics, proteomics	Genome and protein profiling	Genome annotation and prediction	[135]
MS2	*E*. *coli*	Metabolomics	Phage–host interaction	Functional metabolic outcomes of infection	[136]

The integration of transcriptomic and proteomic data enables the identification of critical bacterial processes required for survival during phage infection, thereby allowing the development of phages that preferentially target these pathways with enhanced efficiency and potentially result in faster and more effective bacterial lysis [137]. For example, when transcriptomic analyses reveal the upregulation of stress response genes and proteomic data confirm the presence of corresponding stress‐related proteins, phages can be designed or selected to deliberately interfere with these proteins, consequently compromising bacterial defense mechanisms and enhancing phage efficacy.

Personalized phage therapy has become increasingly feasible through the integration of multiple omics approaches, enabling treatments to be tailored to the unique molecular profiles of bacterial infections in individual patients [138]. By analyzing transcriptomic, proteomic, and metabolomic data from a specific bacterial strain, researchers can design or select phages that are most likely to be effective against it. This customized strategy is crucial for treating infections caused by MDR pathogens, where conventional phage cocktails may be ineffective [139].

Furthermore, integrating data from multiple omics platforms enables the identification of biomarkers that can predict the efficacy of phage therapy. These biomarkers may include specific metabolites, proteins, or gene expression profiles that are associated with bacterial susceptibility to phage infection [54]. For instance, if a particular metabolic pathway is consistently disrupted in bacterial strains that are susceptible to a given phage, the metabolites associated with it could serve as biomarkers to guide phage selection in clinical applications.

Multiomics approaches are also essential for elucidating the mechanisms underlying bacterial resistance to phages [140]. By examining transcriptomic and proteomic changes in bacterial populations that survive phage infection, researchers can identify the genetic and protein‐level adaptations that confer resistance to phage infection. This knowledge can then be leveraged to develop phages capable of circumventing these resistance mechanisms, either by directly targeting newly acquired pathways or by combining phage therapy with adjunct treatments that inhibit the emergence of resistance [23].

### 7.4. Challenges and Prospects

Although multiomics data integration in phage therapy has enormous potential, it also presents distinct problems [141, 142]. Table 3 highlights some methodological challenges in leading bioinformatics tools/pipelines for multiomics integration in microbial systems.

**Table 3:** Summary of the leading bioinformatics tools for multiomics integration in microbial systems.

Tool/pipeline	Omics integrated	Key features	Strengths	Limitations	Reference
mixOmics	Genomics, transcriptomics, proteomics, metabolomics	Multivariate approaches (PLS, sPLS, DIABLO) for integration and feature selection	Strong for biomarker discovery; powerful visualization; widely used	Requires R expertise; computationally demanding for large datasets	[143, 144]
MOFA+	Genomics, transcriptomics, epigenomics, proteomics	Unsupervised factor analysis identifying shared and dataset‐specific variation	Handles missing data, interpretable latent factors, and scalability	Requires statistical knowledge; less intuitive for beginners	[143, 145]
PaintOmics	Transcriptomics, metabolomics (with genomics support)	Pathway‐based integration and visualization using KEGG maps	User‐friendly; strong biological interpretation via pathways	Limited for deep statistical integration; dependent on pathway databases	[146]
iClusterPlus	Genomics, transcriptomics, epigenomics	Joint latent variable model for clustering multiomics data	Good for identifying strain/population structure	Limited metabolomics support; less scalable	[145]
Cytoscape (with plugins)	Flexible (multiomics)	Network‐based integration and visualization	Excellent for interaction networks; highly customizable	Requires plugins for full integration; less automated	[147, 148]
MetaboAnalyst	Metabolomics + transcriptomics (limited multiomics)	Statistical analysis, pathway mapping, and data integration tools	Easy to use; strong metabolomics focus	Limited depth for full multiomics integration	[146, 149]

Also, the vast amount and intricate nature of data necessitate advanced bioinformatics tools and specialized knowledge in data analysis. Furthermore, the task of evaluating the connections among several omics layers can be challenging, since alterations in one layer may not always reflect directly on alterations in another [142, 150]. For example, a gene can exhibit high expression levels, as demonstrated by transcriptomics. However, its associated protein may undergo posttranslational changes that change its function, a specific piece of information that can only be detected using proteomics approaches. Also, observed discrepancies between mRNA and protein levels often suggest regulatory mechanisms at the translational level. Translatomics, such as Ribo‐seq, elucidates transcripts undergoing active translation, whereas phage‐encoded small RNAs (sRNAs) can modify mRNA stability or translation efficiency [151–154]. The interplay among these layers elucidates the reasons for the divergence between transcript and protein data, underscoring the significance of “missing links” essential for comprehending phage–host interactions in the context of multiomics studies [155, 156].

Moreover, comparing different omics metrics for the same biological subsystem poses challenges in the context of multiomics data, complicating the communication of the alterations occurring within a biological system [157].

Continued progress in computational biology, machine learning, and systems biology is anticipated to further enhance the integration and analytical capabilities of multiomics data. These technologies will facilitate the construction of more precise models of phage–host interactions, hence facilitating the advancement of next‐generation phage therapies that exhibit enhanced efficacy, specificity, and individualization [53, 158].

Furthermore, with the ongoing reduction in the expenses associated with omics technologies, it is anticipated that multiomics methods will become more affordable, therefore enabling their regular utilization in phage treatment research and clinical applications [159]. Undoubtedly, this will result in a more profound comprehension of the dynamics between phages and bacteria, thus creating novel opportunities to address antibiotic‐resistant diseases.

Ultimately, the incorporation of multi‐omics data is crucial for fully harnessing the capabilities of phage therapy [160, 161]. Through a thorough analysis of the molecular interactions between phages and bacteria, this method has the potential to facilitate the creation of more efficient, focused, and individualized therapies, thus improving the clinical efficacy of phage therapy [71, 162].

## 8. Conclusion and Future Perspective

Molecular metabolomics provides valuable insights into the metabolic alterations that occur during phage infection, thereby uncovering potential metabolic susceptibilities in bacteria. Transcriptomics reveals the alterations in gene expression that phages cause to undermine bacterial defenses, providing opportunities to improve the effectiveness of phage toxins. In turn, proteomics reveals the protein‐level interactions and PTMs that are crucial for the replication of phages and the response of bacteria. The integration of multiomics methodologies, including those mentioned above, represents a major breakthrough in the field of phage therapy. These various omics techniques provide a robust set of tools for the development of more efficient and individualized phage therapeutics. By integrating these various strata of biological data, scientists can gain a comprehensive understanding of the complex interactions between bacteriophages and their bacterial hosts. It is essential to adopt a comprehensive viewpoint in order to maximize phage therapy, especially considering the increasing phenomenon of antibiotic resistance.

In the future, the ongoing progress in computational tools and the declining expenses of omics technologies are anticipated to enhance the accessibility of multiomics techniques. These developments are expected to lead to further advancements in phage therapy, providing renewed optimism in combating antibiotic‐resistant diseases and making a valuable contribution to the broader field of precision medicine.

## Author Contributions

Conceptualization: D.J.A. and C.N.A. Methodology: D.J.A. Validation: D.J.A. and C.N.A. Writing—original draft preparation: D.J.A. Writing—review and editing: D.J.A. and C.N.A. Supervision: C.N.A.

## Funding

No funding was received for this manuscript.

## Disclosure

All authors have read and agreed to the published version of the manuscript.

## Ethics Statement

The authors have nothing to report.

## Consent

The authors have nothing to report.

## Conflicts of Interest

The authors declare no conflicts of interest.

## Data Availability

Data sharing is not applicable to this article, as no datasets were generated or analyzed during the current study.
